# Late adolescent outcomes of childhood trajectories of internalizing symptoms: an 8-year follow-up of depressive and anxiety symptoms and cognitive, emotion- and behavior-related self-regulation facets

**DOI:** 10.1186/s13034-025-00958-6

**Published:** 2025-08-31

**Authors:** Johanna Lilian Klinge, Petra Warschburger, Annette Maria Klein

**Affiliations:** 1 International Psychoanalytic University Berlin, Stromstr. 1, 10555 Berlin, Germany; 2 Department of Psychology, University of Potsdam, Karl-Liebknecht-Str. 24-25, 14476 Potsdam, Germany

**Keywords:** Self-regulation, Adolescence, Trajectories, Anxiety symptoms, Depressive symptoms

## Abstract

**Background:**

Internalizing symptoms are highly prevalent in childhood and adolescence. Several studies have demonstrated heterogeneity in symptom trajectories and examined their predictors. However, little is known about their outcomes in late adolescence. Building on a previous study that identified a stable low, an increasing and an (early high) decreasing trajectory of internalizing symptoms in childhood, this follow-up study examines their outcomes, including mental health-related measures and self-regulation facets.

**Methods:**

Trajectories of internalizing symptoms were identified using parent reports at three measurement points in a community sample of *N* = 1453 children aged 6–13 years, based on the Emotional Problems Scale of the Strengths and Difficulties Questionnaire. At the 8-year follow-up, *n* = 556 adolescents aged 16–21 years participated, providing self-reports on mental health-related measures and self-regulation facets (emotional reactivity, emotion regulation, planning behavior, risk taking, impulsiveness, delay discounting). Additionally, three self-regulation facets (working memory updating, inhibition, risk taking) were assessed behaviorally.

**Results:**

Adolescents in the increasing trajectory reported significantly greater internalizing symptoms, more specific anxiety symptoms, greater distress and social impairment, and more impaired personality functioning than those in the stable low trajectory, and more specific depressive symptoms than those in the stable low and decreasing trajectories. Regarding SR facets, they reported lower planning behavior and less use of the emotion regulation strategies reappraisal and positive reappraisal than those in the stable low trajectory, and greater use of the emotion regulation strategy catastrophizing than those in the stable low and decreasing trajectories.

**Conclusions:**

Children with increasing internalizing symptoms in childhood present internalizing symptoms and self-regulation deficits in late adolescence that can hinder further development. In contrast, the differences observed in childhood between the decreasing and the stable low trajectories are no longer detectable. Promoting self-regulation could be a promising prevention and intervention target. Future research should investigate protective factors contributing to symptom remission.

**Supplementary Information:**

The online version contains supplementary material available at 10.1186/s13034-025-00958-6.

## Background

Internalizing symptoms include anxiety, depressive and somatic symptoms, and withdrawal. They are among the most common psychological symptoms in children and adolescents [1] with a prevalence of 15.0% each of clinically relevant anxiety and depressive symptoms in seven to 17-year-olds in Germany (BELLA study; [2]). Affecting almost all areas of children's and adolescents' lives (e.g., school, family, peers), internalizing symptoms negatively impact the achievement of later developmental goals (e.g., academic success; [3]) and can become chronic into young adulthood [4]. In a representative German sample, 35.3% of the participating 16- to 24-year-olds presented at least mild depressive symptoms (value ≥ 4 on the Patient Health Questionnaire-9; [5]), and in a random community sample of 14- to 21-year-olds, 23.3% fulfilled the DSM-5 criteria for at least one lifetime anxiety disorder [6]. Compared with boys, girls are at a greater risk of developing internalizing symptoms from the onset of puberty [7].

In the last decade, studies using person-centered approaches have shown that there is interindividual heterogeneity in terms of the onset and stability of internalizing symptoms during childhood and adolescence. Most of these studies focus on depressive symptom trajectories (meta-analysis: [3]; selection of single studies: [8, 9, 10, 11]), whereas there are less studies on anxiety or broader internalizing symptoms (e.g., [12, 13, 14]). The number of identified trajectories usually varies between three and four, differing in terms of the severity (low, medium, high) and stability (stable, increasing/decreasing) of symptoms (e.g., [13, 14, 15, 16, 17, 18, 19]). In these studies, most participants reported stable low or no symptoms, whereas smaller subgroups (usually < 10%) experienced increasing or stable high symptoms.

Understanding the differences between children and adolescents who exhibit adverse trajectories of internalizing symptoms (e.g., increasing or stable high symptoms) and those who do not can help identify individuals at greater risk for negative long-term outcomes. However, despite late adolescence being a critical and vulnerable period characterized by multiple developmental tasks related to independence and autonomy [20], few studies have examined outcomes in this age group. During this period, adolescents undergo significant physical changes and begin to increasingly detach from their parental home. They also form deeper friendships, experience their first romantic relationships, and develop their own values. To navigate these expanding developmental contexts, adolescents increasingly rely on self-regulation (SR), i.e., the ability to control and modulate one’s cognitions, emotions, behaviors, and physiological responses to attain future benefits [21–23]. Given the robust associations demonstrated in several studies between impaired SR facets and internalizing symptoms (e.g., [21]), it is important to investigate SR facets as outcomes of internalizing symptom trajectories, particularly in late adolescence.

In a previous community-based study [16], we identified three trajectories of internalizing symptoms from ages 6–13: (1) *stable low*, (2) *increasing*, and (3) *(early high) decreasing* internalizing symptoms. We also found that several SR facets could differentially predict the increasing and (early high) decreasing trajectories. This study presents a follow-up investigation of the outcomes associated with these three trajectories eight years later, when participants were aged 16–21 years. First, we examine mental health-related outcomes, including internalizing symptoms, specific anxiety and depressive symptoms, impairment and social distress, and personality functioning. Second, we investigate SR facets as outcomes—both those identified as relevant in our previous study (emotional reactivity and cognitive SR facets), and those considered particularly important during late adolescence (e.g., emotion regulation and risk taking).

### Mental health-related outcomes of trajectories of internalizing symptoms

While several studies have investigated predictors of trajectories of internalizing symptoms (e.g., [3]), significantly fewer have examined their outcomes. Most of these studies focus on psychological symptoms or other psychosocial variables assessed in early adulthood. They consistently indicate that members following increasing or stable high trajectories of internalizing or depressive symptoms are more likely to have mental health problems [8, 11], diagnoses of depressive disorders (meta-analysis: [24]; single studies: [9, 12], or of anxiety disorders [9, 10] in early adulthood than those following stable low trajectories. These differences, however, are not evident between members with decreasing and stable low trajectories [11, 12].

Another mental health-related dimension that is currently gaining importance in scientific discourse is personality functioning. Personality functioning, as defined by the Operationalized Psychodynamic Diagnosis (OPD; [25]), encompasses core psychological functions related to the self (e.g., identity perception, impulse control, self-regulation) and to others (e.g., relationship regulation, empathy, attachment) which begin to develop during early parent-infant interactions [25]. The concept is closely related to personality dysfunction, a dimensional construct used as Criterion A in the Alternative Model of Personality Disorder of the DSM-5, which was recently adopted in the ICD-11 to assess the severity of personality disorders [26, 27]. Consistent with this, adolescents with personality disorders exhibit greater impairments in personality functioning than their healthy peers [28]. Moreover, impairment in personality functioning is associated with depressive and anxiety symptoms [29] and has been shown to mediate the relationship between childhood maltreatment and both depressive [30] and somatic symptoms [31]. Thus, personality functioning represents an important—and according to OPD potentially transdiagnostic—dimension of mental health. Given the conceptual overlap between several aspects of personality functioning and SR, the construct also serves as a bridging concept between mental health-related outcomes and the additionally investigated SR facets.

### Self-regulation facets

SR is a multidimensional construct whose individual facets differ in terms of the developmental phase in which they emerge, their developmental trajectory, and their relationship to other SR facets (for a review, see [23]). To account for the multidimensionality of SR, in the present study, we include cognitive facets (working-memory updating, inhibition, planning behavior), emotion-related facets (emotional reactivity, emotion regulation strategies), and behavior-related facets (delay discounting, impulsivity, risk taking).

Working memory updating (i.e., the ability to mentally retain and manipulate information), and inhibition (i.e., the ability to suppress primary behavioral impulses in favor of a less dominant response) are considered early developing core executive functions (EFs), from which higher-order EFs as planning behavior (i.e., skills such as goal setting, strategy development, and action organization) emerge [32, 33]. Emotional reactivity refers to emotional responses to events in terms of response threshold, latency, amplitude, rise time to peak intensity, and recovery time [34]. In contrast, emotion regulation can be defined as processes that serve to control and modify, whether, when, and how individuals experience and express emotions and emotion-related motivational and physiological states [35]. Emotion regulation strategies are often distinguished as being either adaptive or maladaptive (review: [36]). Delay discounting (i.e., the ability to postpone an immediate, smaller reward in favor of a larger, delayed reward; [37]) is closely related to impulsivity (i.e., the tendency to act without prior planning or strategic consideration, and with insufficient regard for consequences; [38]). Risk taking refers to engaging in behaviors that involve the potential for danger or harm, while also offering the possibility of a reward [39].

### Self-regulation facets as outcomes of trajectories of internalizing symptoms

A growing body of research shows associations between lower SR facets and internalizing symptoms during childhood and adolescence (e.g., [21]). However, the directionality of the relationship between SR facets and internalizing symptoms remains unclear. Multiple studies have demonstrated that lower SR facets increase the risk for the development of internalizing symptoms, a notion supported also by meta-analyses linking lower executive functions [40] and negative affectivity [41] in childhood with later internalizing symptoms. However, initial longitudinal studies using cross-lagged panel models have also demonstrated bidirectional associations between SR facets and internalizing symptoms—that is, internalizing symptoms predict later SR deficits and vice versa (e.g., [42])—as well as temporally stable between-person effects [43–45]. These findings suggest that SR and internalizing symptoms may conceptually overlap and codevelop along a shared continuum (for a detailed discussion, see [44]). Given the different explanatory models regarding the associations of SR facets and internalizing symptoms during development, it is crucial to examine SR facets not only as predictors but also as outcomes of internalizing symptom trajectories.

To date, only a few studies have investigated SR facets as adolescent outcomes of childhood trajectories of internalizing symptoms. Most of them have examined risk-taking behaviors or risky lifestyle factors. These are partially related to the behavioral SR facet of risk taking which is investigated in the present study. One study found that girls in an increasing internalizing trajectory reported greater cigarette and cannabis use at age 14 than those in a low trajectory, as well as higher alcohol use than those in a moderate trajectory [13]. Boys with increasing or high internalizing trajectories reported greater cigarette use than those with a low trajectory, with no group differences in alcohol or cannabis use. Another study reported that adolescents who followed an increasing or moderately high depression trajectory had a greater prevalence of risky lifestyle factors in young adulthood (e.g., committing crimes or smoking) than those in the stable low trajectory [46]. However, risk-taking behaviors and other behavior-related SR facets have generally been more strongly associated with externalizing disorders than with internalizing disorders [47, 48].

To the best of our knowledge, no prior studies have investigated emotional and cognitive SR facets as outcomes of internalizing symptom trajectories. Based on previous research, emotion-related facets may be more strongly linked to internalizing symptoms during adolescence. Studies consistently show that greater use of maladaptive emotion regulation strategies and less use of adaptive emotion regulation strategies are associated with depressive and anxiety symptoms (review: [36]; meta-analysis: [49]). Additionally, case–control studies have shown that adolescents with anxiety disorders experience more intense and frequent negative emotional reactions, reflecting heightened emotional reactivity [50]. Compared with healthy controls, adolescents with depressive disorders report higher levels of daily negative affect and lower levels of positive affect [51]. With respect to cognitive SR facets, a meta-analysis revealed that cognitive control appeared to be impaired in middle-aged or older adults with clinical depression but not in children, adolescents, or young adults with clinical depression compared with controls [52]. This aligns with findings from meta-analyses, which report deficits across all EF domains in adults with depression (e.g., [53]), whereas no deficits could be found in inhibition, set-shifting or planning in depressed youth [54].

### Research gaps

As previously outlined, most research using person-centered approaches has focused on trajectories of depressive symptoms, while fewer studies have examined trajectories of anxiety or broader internalizing symptoms. Additionally, person-centered approaches have predominantly focused on identifying predictors of internalizing symptom trajectories, rather than examining their outcomes. When outcomes are investigated, they have largely been limited to psychological symptoms or psychosocial variables, with little attention given to SR facets, despite growing evidence linking reduced SR to internalizing symptoms and broader psychopathology. Furthermore, outcomes have typically been assessed in adulthood rather than during late adolescence—a critical and vulnerable period characterized by numerous developmental challenges and the transition to autonomy [20]. Finally, existing studies often rely solely on either parent-reported or self-reported data, although studies have shown that parent reports tend to yield lower scores when compared to self-reports during adolescence [55]. Only a few studies (e.g., [19]) begin in childhood—typically relying on parent reports of internalizing symptoms—and subsequently examine whether these trajectories differ in later outcomes reported by the adolescents themselves.

### Current study and hypotheses

To address these research gaps, this study builds on a previous community-based study [16] in which we identified three trajectories of internalizing symptoms—stable low, increasing, (early high) decreasing—during middle childhood (aged 6–13 years, assessed from 2012 to 2015) based on parent reports. The present study examines various outcomes of these trajectories at a follow-up 8 years after the last measurement (aged 16–21 years, assessed from 2022 to 2024). Outcomes included self-reported mental health-related outcomes (internalizing, anxiety and depressive symptoms, distress and social impairment, personality functioning) which we examined to test the continuity of internalizing symptoms after an 8-year interval and to examine whether parent-reported symptom ratings in middle childhood correspond to later self-reports of internalizing symptoms. To investigate this further, we also examined the correlation between self- and parent reports of internalizing symptoms in adolescence. In addition to these mental health-related outcomes, we examined cognitive, emotion-related, and behavior-related SR facets as outcomes of internalizing symptom trajectories. Some SR facets were assessed using self-reports (emotional reactivity, emotion regulation strategies, planning, delay discounting, impulsivity) while others were assessed behaviorally (working memory updating, inhibition). One SR facet (risk taking) was assessed using self-reports and behaviorally.

First, we expect participants following the increasing trajectory during middle childhood to exhibit more severe internalizing, depressive and anxiety symptoms, more distress and social impairment, and more impairments in personality functioning in late adolescence than participants following the stable low or the decreasing trajectories. Second, we expect participants following the increasing trajectory during middle childhood to show lower levels of SR in late adolescence compared to participants following the stable low or the decreasing trajectories. The second hypothesis applies primarily to SR facets that have been shown to be associated with internalizing symptoms during adolescence, namely emotional reactivity and emotion regulation strategies. The remaining SR facets are investigated exploratively.

## Methods

### Sample and procedure

Data were collected in a large community-based longitudinal study on intrapersonal developmental risk factors in childhood and adolescence conducted at the University of Potsdam, Germany (for an overview, see study protocol [56]). Participants and their families were recruited from 120 classes in 33 public primary schools in the Federal State of Brandenburg. For an overview of the sample sizes, age and gender distributions at all measurement points see Table 1.

**Table 1:** Overview of the sample sizes, age and gender distributions at all measurement points

	*N*_children_ (%)	*N*_parents_ (%)	*M*_Age_	*SD*	Age_Min_	Age_Max_	girls (%)
t1 (2011/2012)	1657 (100)	1340 (80.9)	8.36	0.95	6	11	52.2
t2 (2012/2013)	1612 (97.3)	1197 (72.2)	9.11	0.93	7	11	51.9
t3 (2014/2015)	1534 (92.6)	1070 (64.6)	11.06	0.92	9	13	51.7
t4 (2022–2024)	570 (34.4)	457 (27.6)	18.88	1.05	16	21	54.4

Participation in the study was voluntary. At t1, t2, and t3, parents gave informed consent. At the follow-up t4, the adolescents provided their informed consent, and parental consent was also obtained for participants who were underage. Assessments were approved by the Research Ethics Board at the University of Potsdam.

Data from t1 to t3 were analyzed in a previous study [16]. This study focuses on follow-up data collected at t4. During a two-hour guided online session, both self-reported and behavioral data were collected from adolescent participants. They received a compensation of €20, plus performance-based incentives from two behavioral tasks (mean: €5.78). Additionally, a 30-min in-person session was conducted to administer behavioral measures that could not be completed online; participants received a compensation of €10. Parents completed questionnaires from t1 to t3 either online or in paper–pencil versions without receiving any incentives.

In our previous study, trajectories of internalizing symptoms were calculated based on a subsample of *n* = 1453, which included all individuals whose parents had reported on their child’s internalizing symptoms at least once from t1–t3. Of these, *n* = 556 adolescents participated again at t4. Based on these two subsamples, participants who took part again at t4 did not differ significantly in internalizing symptoms at t3 from those who did not participate (*t*(1031) = 1.29, *p* = 0.199). However, they differed significantly in terms of age at t3 (*t*(1356) = 2.74, *p* = 0.006, *d* = 0.15), parental income level at t3 (*t*(1019) = − 7.42, *p* < 0.001, *d* = − 0.46), gender at t3 (${\rm X}^{2}$ = 6.15, *p* = 0.013, *Cramer’s V* = 0.07), and membership in trajectory classes from t1 to t3 (${\rm X}^{2}$ = 7.35, *p* = 0.025, *Cramer’s V* = 0.07). The participants at t4 were younger, had a higher parental income, were more often female and more often belonged to the stable low trajectory compared with the dropouts. The effect sizes for differences in the distribution between genders and trajectory classes, however, indicate very low relevance, especially as chi-square tests quickly become significant with large sample sizes. Additionally, all trajectory classes were of meaningful size at t4, enabling complete case analyses with satisfactory statistical power of 0.89, assuming a minimum detectable effect size of ƞ^2^ = 0.015 and an alpha level of 0.05.

The sociodemographic characteristics of both the final sample (*n* = 556) and the participants belonging to different trajectory classes at t4 are provided in Table 2. Analyses revealed significant differences between trajectory classes in subjective socioeconomic status and subjective education status (assessed by the McArthur Scale; [57]), as well as in experiences with outpatient psychotherapy. Post-hoc tests revealed that members in the stable low trajectory reported a higher subjective socioeconomic status than did those in the increasing trajectory. Members in the increasing trajectory reported more experiences with outpatient psychotherapy than those in the other two trajectory classes.

**Table 2:** Sociodemographic characteristics of the final sample and different trajectory classes at t4

	Total sample	Stable low trajectory	Increasing trajectory	Decreasing trajectory	Difference tests between trajectory classes
*N*	556	477	44	35	
Age, *M* (*SD*)	18.87 (1.05)	18.86 (1.06)	18.59 (1.04)	19.02 (0.94)	*F*(2, 554) = 0.42, *p* = .659, ƞ^2^ = .002
*Gender*, *N* (%)					Fisher’s exact test: *p* = .457
Female	301 (54.1)	255 (53.5)	24 (54.5)	21 (60.0)	
Male	243 (43.7)	213 (44.7)	17 (38.6)	13 (37.1)	
Diverse	12 (2.2)	9 (1.9)	2 (4.5)	1 (2.9)	
Subjective socioeconomic status^1^, *M* (*SD*)	7.19 (1.46)	7.27 (1.43)^a^	6.70 (1.56)^b^	6.66 (1.41)^b^	*F*(2, 553) = 5.69, *p* = .004, ƞ^2^ = .020
Subjective education status^1^, *M* (*SD*)	7.73 (1.47)	7.80 (1.43)	7.32 (1.65)	7.23 (1.65)	*F*(2, 553) = 4.39, *p* = .013, ƞ^2^ = .016
*Migration background*, *N* (%)					Fisher’s exact test: *p* = .007
Born in Germany	552 (99.3)	476 (99.8)	43 (97.7)	33 (94.3)	
Not born in Germany	4 (0.7)	1 (0.2)	1 (2.3)	2 (5.7)	
*Parents’ migration background*, *N* (%)					Fisher’s exact test:* p* = .407
Both born in Germany	509 (91.5)	438 (91.8)	38 (86.4)	33 (94.3)	
One not born in Germany	35 (6.3)	29 (6.1)	5 (0.9)	1 (0.2)	
Both not born in Germany	11 (2.0)	9 (1.9)	1 (2.3)	1 (2.9)	
*Current employment*, *N* (%)					Fisher’s exact test:* p* = .654
School	197 (35.4)	165 (34.6)	16 (36.4)	12 (34.3)	
University	159 (28.6)	134 (28.1)	9 (20.5)	9 (2.6)	
Apprenticeship	68 (12.2)	54 (11.2)	9 (20.5)	2 (5.7)	
Work	36 (6.5)	33 (6.9)	1 (2.3)	2 (5.7)	
Else (e.g., voluntary work, internship)	86 (15.5)	71 (14.9)	7 (15.9)	8 (22.9)	
Looking for work	24 (4.3)	20 (4.2)	2 (4.5)	2 (5.7)	
*Experience with psychotherapy*^2^					
Outpatient psychotherapy	134 (24.1)	102 (21.4)	19 (43.2)	13 (37.1)	${\rm X}^{2}$(2, *N* = 556) = 13.94, *p* < .001
(Partial-) Inpatient psychiatric treatment	20 (3.6)	14 (2.9)	4 (9.1)	2 (5.7)	Fisher’s exact test:* p* = .066

If cell frequencies were < 5, Fisher’s exact test with Montecarlo simulation (10,000 iterations) was used

^a,b,c^Different letters indicate significant group differences found in post hoc tests (Bonferroni)

^1^The subjective socioeconomic and education status was assessed by the McArthur Scale [56]. Participants were asked to indicate where they placed themselves and their families in comparison to other people in Germany, using an 11-point graphical scale in the form of a ladder (0 = *lowest status* to 10 = *highest status*)

^2^Experience with psychotherapy was assessed on a dichotomous scale (0 = *no*, 1 = *yes*). Participants were asked whether they had used or were currently using outpatient psychotherapy or (partial) inpatient psychiatric treatment

### Measures

The trajectory classes of internalizing symptoms were based on parent reports collected from t1 to t3. Internalizing symptoms at t4 were assessed using self- and parent reports. Further outcome data at t4 were obtained through adolescent self-reports (questionnaires) and behavioral assessments.

#### Mental health-related outcomes

Internalizing symptoms were measured using the 5-item Emotional Problems scale of the Strengths and Difficulties Questionnaire (SDQ; [58]). The items were rated on a 3-point scale (0 = *not true* to 2 = *certainly true*) and summed to create a scale score. Sum scores from parent reports ranging from 0 to 3 are classified as “close to average”, scores of 4 as “slightly raised”, scores of 5 to 6 as “high” and scores of 7 to 10 as “very high” [59]. Sum scores from self-reports ranging from 0 to 4 are classified as “close to average”, scores of 5 as “slightly raised”, scores of 6 as “high” and scores of 7 to 10 as “very high” [59]. The reliabilities for the parent-reported scale at t1- t4 were α = 0.66–0.81 and those for the self-reported scale at t4 were α = 0.79.

Depressive symptoms were measured using the 8-item Patient Health Questionnaire (PHQ-8; [60]). The items were rated on a 4-point scale (0 = *not at all* to 3 = *nearly every day*). Sum scores of 0–4 indicate no, 5–9 mild, 10–14 moderate, 15–19 moderately severe, and 20–24 severe depressive symptoms. The reliability was α = 0.84.

Anxiety symptoms were measured using the 7-item Generalized Anxiety Disorder Scale (GAD-7; [61]). The items were rated on a 4-point scale (0 = *not at all* to 3 = *nearly every day*). Sum scores of 0–4 indicate no, 5–9 mild, 10–14 moderate, and 15–21 severe anxiety symptoms. The reliability was α = 0.87.

Distress and social impairment were measured using the Impact Supplement of the SDQ [58]. The participants first answered a dichotomous item asking whether they experienced difficulties (regarding emotions, concentration, behavior, or relationships). If they responded affirmatively, they then completed four additional items assessing the extent to which these difficulties caused distress and/or interfered with their everyday life (at home, with friends, learning, and leisure activities). These four items could be rated on a 3-point scale (0 = *not at all/only a little* to 2 = *a great deal*). A total impact score was calculated as the sum of the scores of the four items.

Personality functioning was measured using the 12-item short version of the Structure Questionnaire of the Operationalized Psychodynamic Diagnosis (OPD-SQS; [27]). The items were rated on a 5-point scale (1 = *fully disagree* to 5 = *fully agree*). A mean score was calculated, with higher values indicating greater impairments in personality functioning. This scale was assessed as an optional questionnaire provided by a subsample of *n* = 465. The reliability was α = 0.87.

#### Self-reported SR facets

Planning behavior was measured using 5 items of the planning/organizing scale of the Behavior Rating Inventory of Executive Function (BRIEF; [62]). The items were rated on an adapted 4-point scale (1 = *rarely*/*never* to 4 = *almost always/always*). A mean score was calculated, with higher values indicating better planning behavior (higher SR). The reliability was α = 0.72.

Emotional reactivity was measured using the 10-item emotional control scale of the BRIEF [62]. Items were rated on an adapted 5-point scale (1 = *never* to 5 = *always*). A mean score was calculated, with higher values indicating higher emotional reactivity (lower SR). The reliability was α = 0.91.

Delay discounting was measured using the global discounting rate *k* in 28 hypothetical decision questions in which participants could choose between smaller immediate and larger delayed rewards [37]. *K* is the geometric mean of three discounting rates, which are calculated separately for small, medium and large delayed rewards. As an approximation, the *k* values can be interpreted as a percentage decrease in reward per day, i.e., higher values indicate higher delay discounting (lower SR). To ensure a normal distribution, the variable *k* was logarithmically transformed prior to the analyses. High internal consistency was reported for the global discounting rate [37].

Impulsiveness was measured using the 5-item non-planning impulsiveness scale of the short version of the Barrat Impulsiveness Scale (BIS-15; [38]). The items were rated on a 4-point scale (1 = *rarely*/*never* to 4 = *almost always/always*) and inverted to calculate a mean score, with higher values indicating greater impulsiveness (lower SR). The reliability was α = 0.81.

Self-reported risk taking was measured using 2 items (“I am willing to take risks”, “I am happy to take chances”) of the Urgency, Premeditation (lack of), Perseverance (lack of), and Sensation seeking Impulsive Behavior Scale-8 (UPPS I-8; [63]). The items were rated on a 5-point scale (1 = *does not apply at all* to 5 = *applies completely*). A mean score was calculated, with higher values indicating higher risk taking (lower SR). The reliability was α = 0.91.

#### Emotion regulation strategies

Emotion regulation strategies were measured using the Emotion Regulation Questionnaire (ERQ; [64]) and the Cognitive Emotion Regulation Questionnaire (CERQ; [65]). The ERQ comprises a 6-item scale for the adaptive emotion regulation strategy reappraisal and a 4-item scale for the maladaptive strategy suppression. Both scales were rated on a 7-point scale (1 = *strongly disagree* to 7 = *strongly agree*). The CERQ consists of 9 scales that assess different emotion regulation strategies: 5 adaptive strategies (positive refocusing, planning, positive reappraisal, putting into perspective, and acceptance) and 4 maladaptive strategies (self-blame, other-blame, rumination and catastrophizing). Each scale contains 3 items, which are rated on a 5-point scale (1 = *(almost) never* to 5 = *(almost) always*). For each scale of the ERQ and CERQ, a mean score was calculated, with higher values indicating greater use of the respective strategy. The reliability was α = 0.68–0.84.

#### Behaviorally assessed SR facets

Working memory updating was measured by the sum of correctly answered sequences (max. 16) in the digit-span backwards task (ZN-R) from of the German version of the Wechsler Adult Intelligence Scale (WAIS-IV; 66). In this task, participants were asked to repeat digit-spans backwards, increasing the span lengths from 2 to 8 in a maximum of 8 trials, each consisting of two equal-length sequences. The test was stopped when a participant incorrectly repeated both sequences during one trial. The task shows good reliability [66].

Inhibition was measured using the interference score from the Stroop task, based on reaction time (in milliseconds) in 3 blocks of different conditions (neutral, congruent, incongruent) and a total of 126 trials [67]. Higher values of the interference score indicate lower inhibition (lower SR). The task shows a good reliability [67].

Behaviorally assessed risk taking was measured by the riskiness index in 2 blocks of 10 trials of the Balloon Analogue Risk Task (BART; [68]). This task models a real-world scenario involving risky behavior, where taking risks up to a certain threshold leads to rewards, but exceeding that threshold results in negative consequences. In each trial, the participants inflate a balloon that can hypothetically be inflated a maximum of 128 times and that bursts at a pseudo-randomized time (*M* = 64, *SD* = 28). Each pump collects 0.5 cents, which are paid out to them at the end if they stop pumping before the balloon bursts. If the balloon bursts, the money collected in the trial up to that point is lost. The riskiness index is measured by the average number of pumps in trials in which the balloon did not burst. A higher riskiness index indicates greater risk taking (lower SR). The task shows a good reliability [68].

### Analyses

Three trajectory classes of internalizing symptoms (t1–t3) were identified using growth mixture modelling as described in more detail in a previous study [16]. Based on these analyses, this study investigates differences between trajectory classes regarding various outcomes at t4. We conducted four multivariate analyses of covariance (MANCOVAs) followed by univariate analyses of covariance (ANCOVAs) to determine whether trajectory classes significantly differ in (1) mental health-related measures, (2) self-reported SR facets, (3) emotion regulation strategies, and (4) behaviorally assessed SR facets. We included age, gender, and parental income level at t3 as covariates as these variables were significantly associated with dropout from t3 to t4. The inclusion of covariates led to a reduction in *N*, as parental income level at t3 was reported by only a subsample providing parent reports. Therefore, the results of the analyses conducted without the covariates age, gender and parental income level at t3 are presented in Table S1 (see Additional file 1) for comparison purposes.

If Levene’s test indicated a violation of the assumption of homogeneity of variances, Welch’s F test was additionally used to assess statistical significance. To account for alpha error-accumulation, Bonferroni-corrected post-hoc tests were conducted to examine specific differences between trajectory classes. Partial η^2^ was used as the effect size, with values of 0.01 indicating small effects, 0.06 indicating moderate effects and 0.14 indicating large effects [68].

## Results

**Fig. 1:**
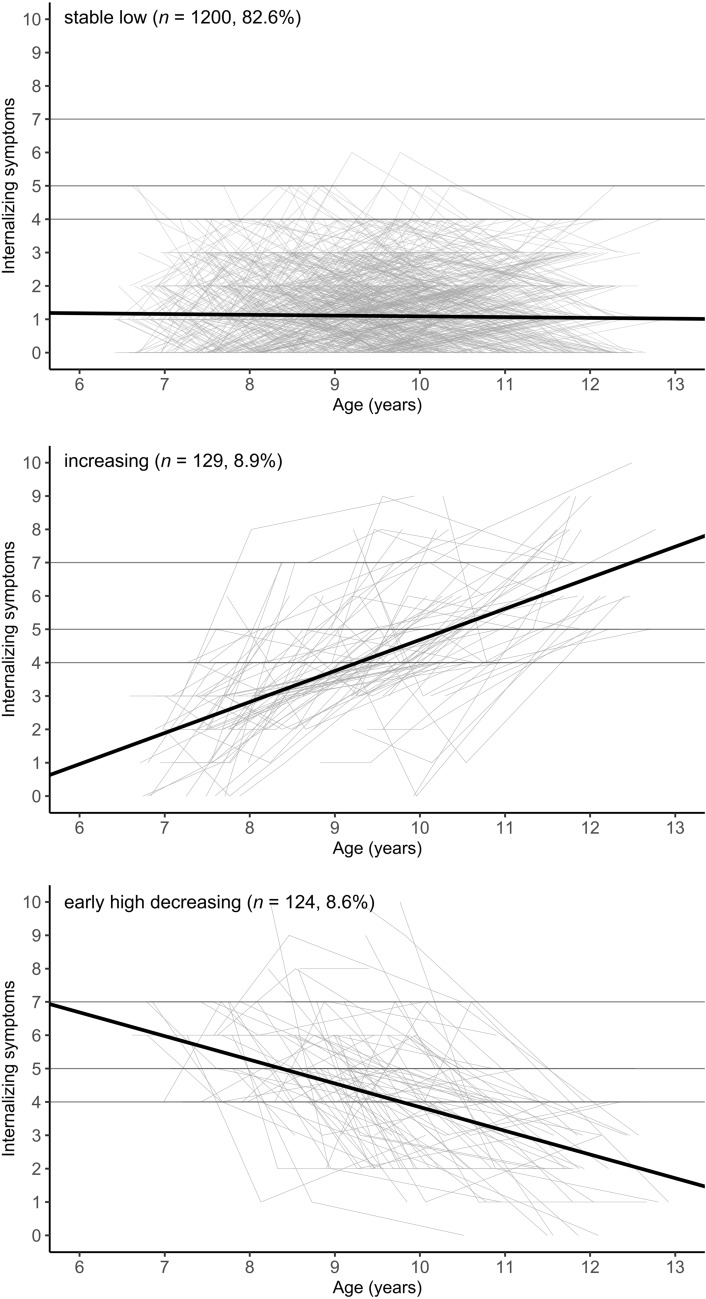
Latent trajectory classes of internalizing symptoms from t1 to t3*.* Black lines represent the latent average trajectory of the respective class, grey lines represent individual trajectories of participants belonging to the respective classes, horizontal lines represent SDQ cut-offs for internalizing symptoms (4 = slightly raised, 5 = high, 7 = very high)

Bivariate correlations of all variables examined are presented in Table S2 (see Additional file 1). The three trajectory classes of internalizing symptoms from t1-t3 are presented in Fig. 1.

### Mental health-related outcomes

The MANCOVA revealed significant differences between trajectory classes in mental health-related measures at t4 (*F*(16, 748) = 2.08, *p* = 0.008, ƞ_p_^2^ = 0.043). In the following ANCOVAs, we found significant differences in all variables (ƞ_p_^2^ = 0.032–0.055). Post-hoc tests revealed that adolescents in the increasing trajectory reported greater internalizing and anxiety symptoms, more distress and social impairment, and more impairments in personality functioning than adolescents in the stable low trajectory. Adolescents in the increasing trajectory reported higher depressive symptoms than adolescents in both the stable low and the decreasing trajectories. For *M*, *F*,* p*, and ƞ_p_^2^ see Table 3. Furthermore, self- and parent-reported internalizing symptoms at t4 were highly correlated (*r* = 0.51, *p* < 0.001).

**Table 3:** Differences between trajectory classes in mental health-related outcomes, self-reported SR facets, emotion regulation strategies, and behaviorally assessed SR facets (t4, M_age_ = 18.87)

	Stable low trajectory	Increasing trajectory		Decreasing trajectory		Univariate tests	
	M (SD)	M	(SD)	M	(SD)	F	ƞ_p_^2^
*Mental health-related outcomes*							
Internalizing symptoms (SDQ)	4.00 (2.64)^a^	6.12 (2.30)^b^		4.92 (2.60)^a,b^		10.81***	.054
Depressive symptoms (PHQ-8)	6.93 (4.58)^a^	10.94 (5.06)^b^		7.88 (5.36)^a^		11.18***	.055
Anxiety symptoms (GAD-7)	5.27 (4.60)^a^	8.67 (4.85)^b^		6.32 (5.63)^a,b^		11.26***^1^	.042
Distress and social impairment (SDQ)	1.16 (1.72)^a^	2.27 (1.93)^b^		1.56 (1.78)^a,b^		6.38**	.032
Personality functioning (OPD-SQS)	31.72 (9.14)^a^	38.12 (8.04)^b^		33.80 (10.73)^a,b^		7.61***	.038
*Self-reported SR facets*							
Planning behavior (BRIEF)	3.08 (0.54)^a^	2.80 (0.66)^b^		2.98 (0.59)^a,b^		5.16**	.022
Emotional reactivity (BRIEF)	2.42 (0.77)	2.72 (0.76)		2.50 (0.74)		2.92^+^	.012
Delay Discounting (DDT)^2^	.014 (0.03)	.012 (0.01)		.009 (0.01)		0.11	< .001
Impulsivity (BIS)	2.24 (0.64)	2.33 (0.55)		2.49 (0.67)		2.52^+^	.011
Risk taking (I-8)	3.47 (0.94)	3.15 (1.10)		3.52 (0.92)		2.14	.009
*Emotion regulation strategies*							
Positive refocussing (CERQ)	3.61 (0.89)	3.31 (1.05)		3.48 (0.84)		0.97	.004
Planning (CERQ)	3.61 (0.89)	3.31 (1.05)		3.48 (0.84)		2.23	.010
Positive reappraisal (CERQ)	3.02 (1.06)^a^	2.33 (1.06)^b^		2.72 (1.00)^a,b^		8.45***	.035
Putting into perspective (CERQ)	3.24 (0.98)	3.05 (1.01)		3.41 (0.82)		1.20	.005
Acceptance (CERQ)	3.62 (0.90)	3.48 (0.91)		3.61 (0.80)		0.47	.002
Reappraisal (ERQ)	4.38 (1.05)^a^	3.90 (1.17)^b^		4.19 (0.95)^a,b^		4.03*	.017
Self-blame (CERQ)	2.73 (0.96)	3.01 (0.98)		2.86 (1.02)		1.68	.007
Other-blame (CERQ)	1.89 (0.63)	1.86 (0.74)		1.69 (0.36)		1.41	.006
Rumination (CERQ)	3.07 (0.94)	3.02 (0.74)		2.96 (0.94)		0.22	.001
Catastrophizing (CERQ)	2.02 (0.80)^a^	2.44 (0.91)^b^		1.97 (0.69)^a^		5.09**	.021
Suppression (ERQ)	3.72 (1.14)	4.14 (1.39)		3.53 (1.03)		2.93^+^	.012
*Behaviorally assessed SR facets*							
Working memory updating (ZNR)	8.55 (1.80)	8.16 (2.23)		8.39 (1.66)		0.81	.004
Inhibition (Stroop)	375.22 (111.77)	388.97 (118.27)		396.82 (159.87)		0.69	.003
Risk taking (BART)	27.08 (11.69)	23.57 (12.72)		24.35 (10.35)		2.07	.010

Covariates age, gender, and parental income level at t3 were included in all analyses, reaching no significance

^a,b,c^Different letters indicate significant group differences found in post hoc tests (Bonferroni)

^1^If homogeneity of variance assumption was not met, we additionally used Welch’s F test to verify significance, revealing the same results

^2^Mean scores and standard deviations are based on the discount rate *k*, while MANOVA, ANOVA and post-hoc tests were performed based on the logarithmically transformed discount rate to ensure normal distribution

^+^*p* < .10, *p < .05, **p < .01, ***p < .001

### Self-reported SR facets

The MANCOVA revealed significant differences between trajectory classes in self-reported SR facets at t4 (*F*(16, 918) = 1.81, *p* = 0.026, ƞ_p_^2^ = 0.031). In the ANCOVAs, we found significant differences in planning behavior (ƞ_p_^2^ = 0.022), marginally significant differences in emotional reactivity and impulsivity, and no significant differences in delay discounting and risk taking. Post-hoc tests revealed that adolescents in the increasing trajectory reported lower planning behavior than adolescents in the stable low trajectory, whereas the other trajectory classes did not differ. For *M*, *F*,* p*, and ƞ_p_^2^ see Table 3.

Notably, in the analyses without covariates, both planning behavior and emotional reactivity were significant (see Table S1 in Additional file 1). Post-hoc tests from the ANOVAs without covariates revealed that members of the increasing trajectory reported greater emotional reactivity than members of the stable low trajectory.

### Emotion regulation strategies

The MANCOVA revealed significant differences between trajectory classes in the use of emotion regulation strategies at t4 (*F*(28, 902) = 1.49, *p* = 0.048, ƞ_p_^2^ = 0.044). In the ANCOVAs, we found significant differences in the use of positive reappraisal (ƞ_p_^2^ = 0.035), catastrophizing (ƞ_p_^2^ = 0.021), and reappraisal (ƞ_p_^2^ = 0.017). Post-hoc tests revealed that adolescents in the increasing trajectory reported less use of positive reappraisal and less use of reappraisal than those in the stable low trajectory. They also reported greater use of catastrophizing than members of both the stable low and the decreasing trajectory. The stable low and decreasing trajectory classes did not differ in any emotion regulation strategies. For *M*, *F*,* p*, and ƞ_p_^2^ see Table 3.

Notably, in the analyses without covariates, the MANOVA did not reach significance (see Table S1 in Additional file 1). However, post-hoc tests from the ANOVAs without covariates revealed significant differences between trajectories in the use of positive reappraisal and catastrophizing, corresponding to the results of analyses with covariates.

### Behaviorally assessed SR facets

The MANCOVA revealed no significant differences between trajectory classes in working memory updating, inhibition, and risk taking at t4 (*F*(12, 844) = 0.89, *p* = 0.561, ƞ_p_^2^ = 0.012). For *M*, *F*,* p*, and ƞ_p_^2^ see Table 3.

## Discussion

In this large community-based prospective study, we investigated late adolescent outcomes of childhood trajectories of internalizing symptoms. Our first hypothesis was partially confirmed, as participants following the increasing trajectory during middle childhood exhibited more internalizing, depressive and anxiety symptoms, more distress and social impairment, and more impairments in personality functioning in late adolescence than participants following the stable low trajectory. They also exhibited more depressive symptoms than participants following the decreasing trajectory, while no further differences between the increasing and the decreasing trajectories were found on other mental health-related measures. Our second hypothesis was also partially confirmed, as participants following the increasing trajectory during middle childhood reported lower planning behavior, and less use of the adaptive emotion regulation strategies positive reappraisal and reappraisal than participants following the stable low trajectory. They also reported greater use of the maladaptive emotion regulation strategy catastrophizing than participants following the stable low or the decreasing trajectory. However, there were only marginally significant differences in emotional reactivity and impulsivity between participants following the increasing trajectory and the stable low trajectory, and no differences in basal cognitive SR facets (working memory updating, inhibition) or behavior-related SR facets (delay discounting, risk taking). There were no significant differences between participants following the decreasing trajectory and the stable low trajectory in any of the investigated SR facets.

### High risk of children with increasing internalizing symptoms until late adolescence

Our study demonstrates that children with increasing internalizing symptoms in middle childhood appear to remain at high risk in late adolescence. Our results are consistent with those of multiple studies reporting that members of increasing trajectories of internalizing or depressive symptoms are more likely to have mental health problems or diagnoses of depression or anxiety disorders in early adulthood than members of stable low trajectories [8–12, 24]. In addition to demonstrating the continuity of symptoms, we also showed that internalizing symptom scores reported by parents in middle childhood corresponded with participants’ self-reports in late adolescence. This was further confirmed by the correlation of *r* = 0.51 between self-reports and parental reports of internalizing symptoms at t4, indicating a moderate to strong level of agreement between the two sources. According to the SDQ cutoffs, participants in the increasing trajectory, on average, reported high internalizing symptoms [59] and scored near or above the clinical threshold of 10 on the PHQ-8 and GAD-7, indicating potential depression or anxiety disorders [60, 61]. Consistently, they also showed greater impairments in personality functioning and had more experience with psychotherapy than members of the stable low trajectory, indicating heightened vulnerability to mental health difficulties.

Furthermore, members of the increasing trajectory exhibited lower levels of two SR facets, planning behavior and emotion regulation, compared to those in the stable low trajectory. Given the limited number of studies examining planning behavior in relation to internalizing symptoms, this finding is particularly noteworthy, especially as the largest effect size was observed for this SR facet. Planning behavior is of particular importance during the period from late adolescence to emerging adulthood, as it is essential for navigating key developmental tasks such as completing school, choosing and starting a career or further education, leaving the parental home, and achieving financial and emotional autonomy [20]. Thus, we assume that members of the increasing trajectory may face greater challenges in transitioning to independent living due to impaired planning behavior. The large effect size underscores the urgent need to support children with increasing internalizing symptoms in developing and strengthening effective planning behavior.

The results on emotion regulation are consistent with several studies showing that the use of adaptive emotion regulation strategies is negatively associated with depressive and anxiety symptoms, whereas the use of maladaptive emotion regulation strategies is positively associated with depressive and anxiety symptoms (review: [36]; meta-analysis: [49]). Similar to planning behavior, the ability to regulate emotions during adolescence is particularly challenged by various developmental tasks in expanding life contexts (e.g. coping with social situations involving peers, university or work). Regarding significant emotion regulation strategies, adolescents in the increasing trajectory seem to have more difficulty reframing situations to experience more positive and fewer negative feelings. They also tend to perceive situations in an overly pessimistic or negative way. Another study, in which emerging adults participated biweekly for eight weeks, also revealed that both depressive and anxiety symptoms were positively associated with catastrophizing and negatively associated with reappraisal at the between-person level [70]. This finding demonstrates that depressive and anxiety symptoms are stably associated with these emotion regulation strategies over time, that is, they tend to co-occur rather than exert a directional influence on one another. It also confirms that reappraisal and catastrophizing may be particularly important in the context of internalizing symptoms and should therefore be considered in prevention and intervention measures.

### Long-term remission of children with decreasing internalizing symptoms until late adolescence?

In contrast to the increasing trajectory of internalizing symptoms, no significant differences were found between the decreasing and stable low trajectories regarding both mental-health related measures and SR facets. These results are consistent with two previous studies reporting no differences in mental health-related outcomes between decreasing and stable low depressive [11] and internalizing symptom trajectories [12]. This suggests that members of the decreasing trajectory may have experienced long-term symptom remission over the course of development. Compared with participants in the increasing trajectory, those in the decreasing trajectory reported significantly lower depressive symptoms and less use of the maladaptive emotion regulation strategy catastrophizing. Descriptively, however, the internalizing, anxiety and depressive symptom levels of adolescents in the decreasing trajectory fall between those of the members in the increasing and stable low trajectories. According to the respective cutoffs of each measure, their internalizing symptoms are slightly raised (SDQ; [59]), and they exhibit mild depressive (PHQ-8; [60]) and mild anxiety symptoms (GAD-7; [61]). Therefore, the nonsignificant group differences may be due to the small group size, and it cannot be ruled out that members of the decreasing trajectory still carry a remaining risk of symptom recurrence later in life.

From a clinical perspective, the decreasing trajectory is particularly noteworthy, as these children exhibited high internalizing symptoms—and thus a high level of risk—in middle childhood, yet they, and potentially their caregivers, were able to reduce these symptoms over time. Given that many children in this group had engaged in psychotherapy, more frequently than those in the stable low trajectory, this may have contributed to the reduction of their symptoms. Investigating potential protective factors possessed by members of decreasing trajectories could be a promising approach to improving prevention and intervention strategies. For example, one longitudinal study examined differences between children who experienced severe childhood stress but developed resiliently versus maladaptively based on their total score on the SDQ, by the time they reached adolescence. In childhood, the resilient group exhibited a more active temperament, greater self-control, and higher cognitive performance compared to the maladaptive group [71]. Other studies have shown that good social skills and secure attachment to parents are negatively associated with later internalizing symptoms [72], suggesting they could be valuable targets for future research.

### Understanding the non-significance of emotional reactivity and basal cognitive and behavior-related SR facets

Unexpectedly, we found no significant differences between trajectory classes in emotional reactivity, or in cognitive and behavior-related SR facets. Below, we discuss the possible conclusions that can be drawn from these findings, taking into account the results of our previous study [16].

#### Emotional reactivity

In our previous study [16], elevated emotional reactivity at the first measurement point was the strongest predictor of membership in the increasing trajectory versus the stable low trajectory. In contrast, we could detect only marginally significant differences between trajectory classes in this variable in late adolescence. Emotional reactivity might be more relevant during early and middle childhood, whereas emotion regulation strategies might be more central in late adolescence. However, the only marginally significant result may also be due to the reduced sample size resulting from the inclusion of covariates, which could have lowered the statistical power. Analyses without covariates revealed significantly greater emotional reactivity in adolescents in the increasing trajectory compared to those in the stable low trajectory, a pattern that is also evident in the descriptive mean values. As many studies have reported moderate to strong associations between emotional reactivity or negative affectivity and internalizing symptoms [41, 50, 51], the marginally significant results should be interpreted with caution.

#### Basal cognitive SR facets

In our previous study [16], basal cognitive SR facets (working memory updating, cognitive flexibility/set-shifting, inhibitory control) at the first measurement point differentially predicted trajectory membership. In contrast, in the present study, no differences between trajectory classes were observed in late adolescence. This finding is consistent with meta-analyses reporting no differences in cognitive control [52] or inhibition and set-shifting [54] in youth with clinical depression versus healthy controls. However, such differences were evident in adults [52–54, 73]. Taken together, these findings suggest that the associations between internalizing symptoms and basal cognitive SR facets may vary across developmental stages, potentially depending on whether these functions are still maturing or have already stabilized. This assumption is supported by findings from a large-scale study [74] reporting that executive functions develop rapidly from ages 10–15 before stabilizing at ages 18–20. In adulthood, pre-existing depressive or anxiety symptoms may also negatively affect previously well-developed SR facets—potentially moderated by the chronicity and severity of the symptoms—or contribute to a more rapid age-related decline in these cognitive functions. A recent study compared 9-month-trajectories of multiple cognitive functions in individuals with recent onset depression and healthy controls, aged 15–40 [75]. Deficits in most cognitive functions were evident at baseline in the depression group compared with the control group. Additionally, the 9-month trajectories of single cognitive functions differed between the depression group and the control group, revealing stable deficits (i.e., same improvement rate in both groups over time), lag (i.e., cognitive impairments of the depression group failed to improve at the same rate as in the control group), and catch-up patterns (i.e., cognitive impairments of the depression group improved at a greater rate than in the control group). Furthermore, cognitive improvements in the depression group were associated with reductions in depressive symptoms. This study’s results indicate that impairments in cognitive functions may be detectable already after the first episode of depression, but follow differential trajectories irrespective of the depressive course, therefore demanding tailored interventions.

#### Behavior-related SR facets

In late adolescence, there were no significant differences in risk taking between internalizing symptom trajectories, as measured by both self-report and behavioral assessment. This contrasts with previous studies linking increasing trajectories of depressive or internalizing symptoms with higher prevalences of risky behaviors or lifestyle factors (e.g., committing crimes, smoking or drug use; [13, 46]). Our results thus demonstrate that adolescents in increasing trajectories of internalizing symptoms may not be impaired in rational risk assessment. Their potential engagement in risky behaviors, as reported in previous studies [13, 46, 76, 77], might instead be driven by alternative mechanisms, such as self-medication through smoking [78]. Finally, behavior-related SR facets may be more strongly associated with externalizing rather than internalizing symptoms [47], which is also reflected in our findings of only marginally significant and non-significant effects for impulsivity and delay discounting.

### Research and clinical implications

In line with the developmental psychopathology framework [79, 80], our findings highlight the importance of considering heterogeneous developmental trajectories of internalizing symptoms from childhood to adolescence. Children who follow an increasing trajectory are particularly at risk of experiencing persistent psychological distress and impairments in personality functioning by late adolescence. As approximately half of mental disorders emerge during childhood and adolescence [81], more effort should be made to identify and support children with prodromal or subclinical symptoms at an early stage. At the same time, it is equally important to investigate the mechanisms that facilitate symptom remission in children following a decreasing trajectory. Understanding protective factors, such as parental sensitivity [82] or parental warmth [83], may offer valuable insights for developing effective prevention strategies.

Based on this evidence, early intervention emerges as a critical priority. Ideally, support should be implemented in middle childhood to prevent symptoms from becoming entrenched. However, if this is missed, interventions in late adolescence are still essential to counteract the long-term negative outcomes associated with chronic internalizing symptoms. Such interventions should not only address emotional distress, but also target deficits in self-regulation by, for example, strengthening planning behavior and fostering the use of adaptive emotion regulation strategies.

### Limitations

While this study has several strengths, including its large community sample, its longitudinal design, and diverse constructs and methods, some limitations should be acknowledged.

First, a significant drop-out occurred between t3 and t4, likely due to the long gap of eight years between assessments and the challenges of recontacting adolescents who had finished school, moved away, or were traveling. In this regard, selection effects were observed, specifically a higher dropout rate among older participants, males, and in those with lower parental income level at t3. However, to avoid dropout-related biases, we controlled for age, gender and parental income level at t3 in our analyses. Furthermore, dropout was not associated with internalizing symptoms at t3 and was only weakly associated with trajectory membership. Also, the remaining group sizes were large enough to provide analyses with sufficient statistical power. Nevertheless, replication in larger samples is warranted to confirm our findings.

Second, the trajectories of internalizing symptoms were derived from a scale consisting of only 5 items. However, this brief SDQ scale has demonstrated good validity, showing high correlations with the more extensive Child Behavior Checklist [84].

Third, we found no differences between trajectories of internalizing symptoms in behaviorally assessed SR facets, including basal cognitive facets and risk taking. As we did not include self-reports of basal cognitive facets, we cannot determine whether the non-significant findings are due to the assessment method. Behavioral measures of executive functions typically measure maximum performance and often show limited overlap with questionnaire-based measures which rather reflect everyday performance [85]. It is possible that questionnaire-based measures might have revealed significant group differences in cognitive SR facets in our study. To verify our findings, future studies should combine both behavioral and questionnaire-based measures.

Fourth, our results cannot be generalized due to the homogeneity of our sample. Further studies should investigate whether our results can be replicated in samples with greater ethnic and socioeconomic diversity to avoid bias in interpretation.

## Conclusion

Our findings highlight the significance of developmental trajectories of internalizing symptoms from middle childhood to late adolescence. Compared with those with stable low symptoms, children who exhibit increasing internalizing symptoms during middle childhood are at greater risk of experiencing persistent internalizing, depressive and anxiety symptoms, distress and social impairments, impaired personality functioning, and difficulties with SR in late adolescence. SR difficulties include lower planning behavior, less use of the adaptive emotion regulation strategies reappraisal and positive reappraisal, and greater use of the maladaptive emotion regulation strategy catastrophizing. In contrast, children whose symptoms decreased during middle childhood did not differ significantly from those with stable low symptoms, suggesting the potential for remission and resilience until late adolescence.

Our findings underscore the importance of middle childhood or, at the latest, adolescence as a critical phase for prevention and intervention. Providing targeted support during this period could mitigate the progression of internalizing symptoms and associated SR deficits, and facilitate better psychosocial outcomes, helping adolescents to navigate essential developmental tasks. Based on our findings, prevention and intervention measures could focus on strengthening planning behavior and promoting adaptive emotion regulation strategies over maladaptive strategies.

## Electronic Supplementary Material

Below is the link to the electronic supplementary material.

Additional file 1

## Data Availability

No datasets were generated or analysed during the current study. Additional file 1: Results of analyses without covariates and Tables S1 and S2.
