# Supplementary material for: Late adolescent outcomes of childhood trajectories of internalizing symptoms: an 8-year follow-up of depressive and anxiety symptoms and cognitive, emotion- and behavior-related self-regulation facets
Source: Child Adolesc Psychiatry Ment Health. 2025 Aug 31;19:101. doi: 10.1186/s13034-025-00958-6 (PMC12400697; doi:10.1186/s13034-025-00958-6)
Supplement: Supplementary file 1 — Additional file 1 [file 13034_2025_958_MOESM1_ESM.pdf]

Supplemental Information for

Late adolescent outcomes of childhood trajectories of internalizing symptoms: an 8-year follow-up of depressive and anxiety symptoms, and cognitive, emotion- and behavior-related self-regulation facets

Klinge, Johanna Lilian[1], Warschburger, Petra[2],

Klein, Annette Maria[1],

[1]International Psychoanalytic University Berlin, Berlin, Germany

[2]Department of Psychology, University of Potsdam, Potsdam, Germany

Results of Analyses Without Covariates

The first MANOVA revealed significant differences between trajectory classes in mental health-related outcomes at t4 (F(10, 890) = 5.45, p = .006, η_p^2 = .027). The second MANOVA revealed significant differences between trajectory classes in self-reported SR facets at t4 (F(10, 1094) = 2.35, p = .010, η_p^2 = .021). The third MANOVA revealed no significant differences between trajectory classes in emotion regulation strategies at t4 (F(22, 1080) = 1.48, p = .071, η_p^2 = .029). The fourth MANOVA revealed no significant differences between trajectory classes in behaviorally assessed SR facets at t4 (F(6, 1004) = 0.97, p = .442, η_p^2 = .006). For M, F, p, and η_p^2 of all conducted ANOVAs see Table S2.

Table S1.

Differences between trajectory classes in outcomes at t4 without including covariates in analyses

	Stable low trajectory	*Increasing trajectory*	*Decreasing trajectory*	Univariate tests	
	M (*SD*)	*M* (*SD*)	*M* (*SD*)	*F*	η_p^2
Mental health-related outcomes					
Internalizing symptoms (SDQ)	4.02 (2.66)[a]	6.03 (2.24)[b]	4.72 (2.63)[a,b]	10.42***	.044
Depressive symptoms (PHQ-8)	7.07 (4.64)[a]	10.59 (5.00)[b]	8.00 (5.43)[a]	9.65***	.041
Anxiety symptoms (GAD-7)	5.42 (4.52)[a]	8.51 (4.60)[b]	6.41 (5.69)[a,b]	7.97***	.034
Distress and social impairment (SDQ)	1.17 (1.74)[a]	2.16 (2.01)[b]	1.55 (1.82)[a,b]	5.69**	.025
Personality functioning (OPD-SQS)	31.92 (9.24)[a]	38.00 (7.66)[b]	32.86 (10.66)[a,b]	7.36***	.032
Self-reported SR facets					
Planning behavior (BRIEF)	3.08 (0.54)[a]	2.80 (0.65)[b]	3.01 (0.59)[a,b]	5.30**[1]	.019
Emotional reactivity (BRIEF)	2.41 (0.76)[a]	2.75 (0.74)[b]	2.48 (0.77)[a,b]	3.96*	.014
Delay Discounting (DDT)[2]	.014 (0.03)	.011 (0.01)	.009 (0.01)	0.82	.003

Impulsivity (BIS)	2.23 (0.63)	2.33 (0.54)	2.42 (0.70)	1.83	.007
Risk taking (I-8)	3.46 (0.63)	3.11 (1.08)	3.54 (0.95)	2.93^{+}	.011
Emotion regulation strategies					
Positive refocussing (CERQ)	2.43 (0.90)	2.33 (1.03)	2.21 (0.81)	1.22	.004
Planning (CERQ)	3.59 (0.89)	3.30 (1.02)	3.54 (0.84)	2.13	.008
Positive reappraisal (CERQ)	2.98 (1.05)[a]	2.37 (1.06)[b]	2.79 (1.02)[a,b]	7.05***	.025
Putting into perspective (CERQ)	3.22 (0.98)	3.08 (1.00)	3.34 (0.82)	0.76	.003
Acceptance (CERQ)	3.61 (0.90)	3.45 (0.88)	3.62 (0.81)	0.67	.002
Reappraisal (ERQ)	4.36 (1.04)	3.98 (1.15)	4.25 (0.90)	2.69^{+}	.010
Self-blame (CERQ)	2.74 (0.96)	2.97 (0.97)	2.84 (0.97)	1.21	.004
Other-blame (CERQ)	1.86 (0.62)	1.83 (0.72)	1.69 (0.38)	1.39	.005
Rumination (CERQ)	3.08 (0.96)	3.06 (0.74)	2.99 (0.93)	0.17[1]	.001
Catastrophizing (CERQ)	2.01 (0.77)[a]	2.39 (0.88)[b]	1.96 (0.65)[a]	5.16**	.018
Suppression (ERQ)	3.75 (1.12)	4.11 (1.37)	3.59 (1.02)	2.48^{+}	.009

Behaviorally assessed SR facets					
Working memory updating (ZNR)	8.56 (1.80)	8.12 (2.24)	8.39 (1.73)	1.17	.005
Inhibition (Stroop)	376.12 (107.94)	388.53 (119.29)	389.67 (148.71)	0.42	.002
Risk taking (BART)	26.85 (11.60)	23.68 (12.37)	24.92 (10.37)	1.71	.007

[a, b, c]Different letters indicate significant group differences found in post hoc tests (Bonferroni).

[1]If homogeneity of variance assumption was not met, we additionally used Welch's F test to verify significance, revealing the same results.

[2]Mean scores and standard deviations are based on the discount rate *k*, while MANOVA, ANOVA and post-hoc tests were performed based on the logarithmically transformed discount rate to ensure normal distribution

$^{+}p < .10$, $^{*}p < .05$, $^{**}p < .01$, $^{***}p < .001$

Table S2. Bivariate correlations between investigated variables at t4

	1	2	3	4	5	6	7	8	9	10	11	12	13	14	15	16	17	18	19	20	21	22	23
**1 INT**	--																						
**2 DEP**	.671***	--																					
**3 ANX**	.690***	.798***	--																				
**4 DSI**	.567***	.673***	.628***	--																			
**5 PF**	.670***	.660***	.649***	.588***	--																		
Self-reported SR facets																							
**6 ER**	.676***	-.578***	.648***	.515***	.611***	--																	
**7 PB**	-.285***	-.397***	-.338***	-.338***	-.331***	-.265***	--																
**8 IM**	.096*	1.62***	.095*	.139***	.145***	.093*	-.381***	--															
**9 DD**	.003	.116***	.101*	.054	.077	.148***	-.052	.064	--														
**10 RT**	-.297***	-.042	-.042	-.056	-.129**	-.156***	-.027	.022	.017	--													
Emotion regulation strategies																							
**11 RF**	-.056	-.098*	-.070	-.131**	-.137**	-.028	.054	-.089*	-.028	.061	--												
**12 PL**	-.233***	-.156***	-.133***	-.214***	-.238***	-.206***	.145***	-.316***	-.092*	.168***	.233***	--											
**13 PR**	-.315***	-.274***	-.231***	-.259***	-.307***	-.226***	.185***	-.277***	-.075	.279	.332***	.483***	--										
**14 PP**	-.064	-.044	-.041	-.136***	-.069	-.055	-.002	-.095*	-.021	-092*	.279***	.278***	.389***	--									

**15 AC**	-.167***	-.035	-.066	-.105*	-.079	-.158***	-.001	-.087*	-.049	.208***	.221***	.327***	.356***	.303***	--									
**16 RE**	-.218***	-.192***	-.181***	-.205***	-.256***	-.201***	.081	-.135**	-.093*	.150***	.433***	.377***	.450***	.257***	.242***	--								
**17 SB**	.466***	.442***	.465***	.339***	.427***	.346***	-.251***	.061	.021	-.109**	-.060	-.078	-.152***	.070	.067	-.111***	--							
**18 OB**	-.046	-.035	-.010	-.013	.001	.068	-.094*	.017	.010	.076	.081	.104*	.045	-.016	.014	.046	-.200***	--						
**19 RU**	.418***	.370***	.398***	.292***	.370***	.379***	-.182***	-.022	.006	-.058	.116**	.203***	.120**	.055	.159***	.123**	.303***	.066	--					
**20 CA**	.439***	.322***	.395***	.312***	.348***	.389***	-.242***	.011	<.001	-.074	.076	-.100*	-.163***	-.130**	-.022	-.106*	.302***	.234***	.346***	--				
**21 SU**	.039	.116**	.027	.105*	.255***	-.109**	-.092*	.044	.001	-.045	-.043	-.087*	-.184***	-.006	.106*	.001	.200***	-.020	-.088*	.031	--			
Behaviorally assessed SR facets																								
**22 WM**	-.052	-.056	-.060	-.027	-.119*	-.095*	-.071	-.001	-.060	.057	.008	.063	.040	.044	.051	.048	.005	.053	-.010	-.055	-.059	--		
**23 IN**	.004	.034	.016	-.006	-.085	-.005	-.070	-.047	-.020	.024	-.018	.067	.023	-.001	.046	.022	.034	.037	.008	-.022	.043	.201***	--	
**24 RT**	-.167***	-.156***	-.134***	-.112**	-.103*	-.192***	-.005	.030	-.075	.097*	-.067	.083*	.044	.002	.073	.020	.019	-.036	-.062	-.069	.061	.056	.082	

Note: Abbreviations are INT = internalizing symptoms, DEP = Depressive symptom.082s, ANX = Anxiety symptoms, DSI = Distress and social impairment, PF = personality functioning, SR = Self-regulation, ER = emotional reactivity, PB = planning behavior, IM = Impulsivity, DD = Delay Discouting, RT = Risk taking, RF = Positive Refocussing, PL = Planning, PR = Positive reappraisal, PP = Putting into perspective, AC = Acceptance, RE = Reappraisal, SU = Self-blame, OB = Other-blame, RU = Rumination, CA = Catastrophizing, SU = Suppression, WM = working memory updating, IN = inhibition.

**p* < .05, ***p* < .01, ****p* < .001.
